# Quality in science communication with communicative artificial intelligence: A principle-based framework

**DOI:** 10.1177/09636625251328854

**Published:** 2025-05-22

**Authors:** Daniel Silva Luna, Irene Broer, Helena Bilandzic, Monika Taddicken, Björn W. Schuller, Martin Bürger

**Affiliations:** University of Augsburg, Germany; Technical University of Braunschweig, Germany; University of Augsburg, Germany; Technical University of Braunschweig, Germany; Technical University of Munich, Germany; University of Augsburg, Germany

**Keywords:** AI guidelines, artificial intelligence, communicative AI, quality framework, science communication

## Abstract

The rapid advancement of communicative artificial intelligence (ComAI) is profoundly impacting science communication, offering new opportunities for easier and more audience-oriented communication. However, it also poses several challenges for its practice. Based on a narrative review of literature on science communication and ComAI quality, this article develops a framework of quality principles for science communication with ComAI. The framework identifies the quality dimensions of scientific integrity, human-centricity, ethical responsiveness, inclusive impact, and governance. We discuss applications of this framework in technology development, practitioner training, guideline development, and quality evaluation. This work aims to foster critical discussions on the normative standards for ComAI use in this field.

The advent of communicative artificial intelligence (ComAI) represents a paradigm shift in science communication (Schäfer, 2023). As these technologies evolve, they may not only influence but potentially become the primary voice of science, underscoring the critical role of researchers and practitioners in crafting a strategic vision for science communication with ComAI and guiding its development (Bockting et al., 2023; Schäfer and Wessler, 2020).

ComAI refers to *automation-based technologies embedded in digital infrastructures and human communicative practices, acting as both mediators in human communication and agents in human–machine relations* (Guzman and Lewis, 2020; Hepp et al., 2023). They have evolved from simple chatbots to sophisticated generative models such as ChatGPT, Gemini, Copilot, and Claude, which can engage in nuanced contextual conversations and perform complex agentic roles (Gabriel et al., 2024).

ComAI is transforming various aspects of science communication by automating tasks that previously required human cognition, expertise and communication skills. These technologies not only augment human-to-human science communication – for instance, aiding science journalists in honing their writing (Dijkstra et al., 2024) – but also assume direct communicative roles, with AI chatbots functioning as science communicators in their own right (Schäfer, 2023). Early evidence suggests that ComAI technologies are being widely adopted by professional science communicators (Henke, 2024) and are already being used by the public for scientific information seeking (Greussing et al., 2025). While ComAI’s human-like communication offers significant advances in interactivity, accessibility, and personalisation, their emergence also raises critical questions about their impact on the quality of science communication.

What constitutes ‘quality’ in science communication is a subject of ongoing debate, encompassing diverse aspects such as scientific accuracy, effectiveness, ethical responsibility, diversity of engagement, and evaluation methods (Fähnrich et al., 2023; Fecher et al., 2023b; Medvecky and Leach, 2019; Olesk et al., 2021; Taddicken et al., 2024). However, current quality frameworks do not adequately address the complexities introduced by ComAI to this field, particularly in relation to issues like ‘hallucinations’ in the representation of science, trust in human-machine-interactions, and the economic implications to professional science communication, among other issues (Schäfer, 2023). The unique capabilities of ComAI require a re-evaluation of existing quality frameworks to provide all stakeholders with the conceptual and normative vocabulary necessary to ensure quality science communication with ComAI.

This article conducts a narrative review of the literature at the intersection of quality science communication and ComAI, addressing the question: *What constitutes quality science communication with ComAI?* We have thematically identified five principles that address the unique challenges posed by ComAI technologies: *Scientific integrity, human-centred communication, ethical responsiveness, inclusive impact*, and *governance*.

Our principles are distinguished from existing AI ethical or quality standards by their focus on ComAI as a concept referring to novel, AI-based technologies capable of communicating with people in natural language, and on how this technology shapes the ‘social conversation around science’ (Trench and Bucchi, 2021: 1). This framework aims to stimulate discussions about ComAI’s implications in science communication, engaging both developers and users to mitigate risks and maximise benefits.

We begin by reviewing the literature on quality science communication and quality ComAI and argue for principlism as a flexible approach suitable for the different contexts where science communication occurs. After presenting our methodology, we explore each principle and its key issues. Our discussion considers broader implications for the design of ComAI tools, the training of science communicators, the development of professional guidelines, and the evaluation of ComAI-mediated science communication. Finally, we reflect on limitations and identify future avenues for research.

## 1. Literature overview

The meaning of quality in communication is inherently normative, involving ideals that shape judgements about what constitutes ‘good’ communicative practices, content, processes and institutional standards (Strömbäck, 2005). Professional norms, cultural values, as well as personal beliefs and worldviews all play a role in determining what is considered ‘quality’ communication – which differs from one context to another. This multiplicity is reflected in science communication, too: Whereas scientists may consider accuracy and comprehensiveness the most important indicators of quality in science communication (Maier et al., 2016), science journalists may prioritise evidence and public relevance (Rögener and Wormer, 2017), and different science communication publics may instead focus on presentation, correctness or entertainment value (Taddicken et al., 2024).

### ‘Quality’ in science communication

Despite the lack of a universally accepted definition, discussions about quality are vital to the maturity of the field of science communication (Bucchi and Trench, 2014). Historically, issues of quality in science communication have been concerned with accuracy and objectivity, especially in the representation of science in the media (Hansen, 2016). More recently, there has been a growing focus on how science communication can effectively engage and resonate with diverse audiences (Kearns, 2021). This shift reflects a move towards dialogue-based science communication, emphasising the importance of considering the needs, interests, and perspectives of the public. This more human-centric view looks at attributes such as presentation style, contextual relevance, and interactivity as markers of quality science communication (Fähnrich et al., 2023; Olesk et al., 2021).

However, focusing solely on the effectiveness of engagement can be problematic as ‘some acts of communication may be morally wrong even if they have good consequences’ (Lamb, 2018 [2017]: 5). As a result, other quality questions have focused on ethics and being ‘good science communicators’ (Medvecky and Leach, 2019: 3). Despite the absence of a unified ethical framework, science communication researchers and practitioners have drawn on an array of normative theories, including Mertonian norms, journalistic standards, bioethical principles, and meta-ethical perspectives, to navigate moral behaviour and ethical dilemmas (e.g. Lamb, 2018 [2017]; Medvecky and Leach, 2019; Priest et al., 2019).

Others have expanded the discussion of quality to encompass inclusive practices and quality governance. Recognising that engagement-based approaches tend to ‘preach to the converted’ (Dawson, 2014), there have been growing calls to think of ‘good’ science communication as that which actively promotes equity and inclusion (Canfield et al., 2020). Concurrently, there has been increased interest in quality management, for example, in terms of how scientific organisations incorporate monitoring, feedback, and ongoing training to uphold and improve quality in their communications (Fecher et al., 2023b).

These evolving debates on ‘good’ science communication reveal recurring themes in the literature that align with and expand upon traditional science communication goals: *scientific integrity* ensures it accurately depicts scientific issues, reflecting the traditional aim of knowledge transfer; *human-centricity* focuses on effective engagement with diverse audiences, echoing the dialogue model’s emphasis on resonance and relevance; *ethical responsibility* addresses the broader societal implications of science communication; and *inclusive impact* promotes equity and broad engagement, building on the participatory model (Trench and Bucchi, 2021). The fifth dimension, *governance*, emerges as a meta-level concern not tied to a specific traditional model, but ensuring that quality is maintained and adapted over time, reflecting the dynamic nature of science communication. This aligns with the ideals of reflective science communication, which emphasise continuous learning, self-examination, and adaptation of communicative practices (Roedema et al., 2022). These five aspects of quality in science communication provide a foundation for examining the unique challenges and opportunities presented by ComAI in this field.

### ‘Quality’ in ComAI

As ComAI systems become increasingly sophisticated and widely adopted, defining and ensuring their quality has become a pressing concern for researchers, developers, legislators, and users alike. While the literature on what makes ‘good’ ComAI is still emerging, we can draw parallels with the quality dimensions discussed in science communication: *integrity, human-centredness, ethical responsibility, inclusivity*, and *governance*.

Integrity of ComAI focuses on discussions regarding its factual accuracy, reliability, and source attribution, among other things. For example, studies have evaluated the factuality of models such as ChatGPT, exposing issues like ‘hallucinations’, plausible-sounding but factually incorrect outputs (e.g. Bang et al., 2023). Researchers have also explored the reliability of ComAI in specific fields like medicine (Johnson et al., 2023) and tasks like translation (Hendy et al., 2023). Similar research has also examined ComAI’s ability to cite sources and distinguish verified facts from speculation (e.g. Walters and Wilder, 2023).

In the realm of quality human-computer interactions, scholars emphasise systems that prioritise human experiences and needs. For example, Shneiderman (2020) advocates for designs that amplify human self-efficacy and creativity while ensuring reliability, safety and trustworthiness. This approach aims to mitigate biases, foster meaningful human–AI interactions, and create conditions for respectful and emotionally resonant relations (Ozmen Garibay et al., 2023).

Ethical considerations form another crucial dimension of ComAI quality discussions. Most approaches emphasise responsible development and use, incorporating goals such as security, privacy, beneficence, non-maleficence, justice, and respect for human autonomy (Floridi et al., 2018; Jobin et al., 2019). Quality ComAI systems, from this view, should not only meet technical performance standards but also align with ethical ideals to ensure they contribute positively to society while minimising potential harms.

Similar debates centre on developing ComAI technologies that are inclusive, for example, in terms of ensuring accessibility for diverse user groups; representing diverse points of views, cultures and languages; and promoting equity (e.g. Shams et al., 2025; Stahl and Eke, 2024). These efforts are crucial for ensuring ComAI systems benefit the broadest possible spectrum of society, fostering greater social participation and reducing the digital divide (Luttrell et al., 2020).

Governance considerations also play a crucial role in ensuring ComAI quality. Emerging guidelines and legislation propose accountability measures and quality monitoring mechanisms (e.g. European Commission, 2024; Hartmann et al., 2024). These frameworks are essential for maintaining and improving ComAI quality as these systems become more widespread and influential (Coeckelbergh, 2020).

While individual dimensions such as accuracy, human-centredness, ethics, inclusivity and governance provide valuable lenses for assessments of ‘good’ ComAI, it is the integration of these aspects that truly defines its quality. This holistic view provides a foundation for developing a definition for quality science communication with these technologies.

### Constructing a ‘quality’ framework built on principles

We used the concept of ‘quality’ to bridge the gap between the different discourses about what makes ‘good’ science communication and ComAI. Based on our review, we define quality science communication with ComAI as

the human-centred and ethically responsive design, implementation, and use of ComAI technologies that accurately and reliably convey scientific information, foster inclusive engagment, and operate withinrobust governance frameworks.

Translating this definition into a practical framework presents significant challenges considering the many contexts where science communication is practised (Gascoigne et al., 2020) and the rapidly evolving nature of ComAI technologies and practices. We therefore take a *principles-based* approach: Principlism^1^ provides an adaptable framework for developing and applying principles to decision-making. Unlike rigid meta-ethical frameworks like deontology, principles^2^ are broad normative statements that navigate the complexities of diverse contexts, serving as heuristics to guide thoughtful consideration of behaviours and their impacts (Beauchamp and Childress, 2001), making them particularly suitable for the varied contexts and goals of science communication (Medvecky and Leach, 2019).

However, critics argue that the broadness of principles can foster ambiguity and inconsistency, potentially diminishing their effectiveness in guiding concrete actions (Munn, 2023). In response, principles are recognised as the first step in a layered approach to governance, which should include specific rules and policies to bridge the gap between high-level aspirations and practical applications (Floridi et al., 2018). More importantly, principles serve a central cultural function, shaping the norms and values within professional communities by promoting common vocabulary that guide the evolution of practices over time (Seger, 2022). This cultural influence underscores the significance of principles not only as guidelines but as catalysts for fostering continuous reflective engagement among practitioners (Roedema et al., 2022). Therefore, while principles alone may not provide all the answers, they lay the groundwork for a dynamic and responsive framework, establishing a foundational mind-set from which more detailed and situation-specific guidelines can be developed (Medvecky and Leach, 2019).

The swift advancement of ComAI has spurred numerous principle-based frameworks designed to guide its development and use in specific communication contexts. Examples include guidelines and best practices for ComAI use in journalism (Becker, 2023), in science education (Long and Magerko, 2020), and academic writing (Buriak et al., 2023). Recognising a similar need within our own field (e.g. Dijkstra et al., 2024), we propose a set of five principles that specifically address the challenges and opportunities that ComAI brings to science communication.

## 2. Methodology

While empirical methods such as surveys and Delphi approaches provide valuable insights (e.g. Fähnrich et al., 2023; Olesk et al., 2021), they are limited by their time-intensive nature, restricted sampling frames, and the difficulty for participants to verbalise tacit knowledge and normative beliefs. This can lead to a lack of analytical depth when exploring the complex, rapidly evolving and interdisciplinary topic of ComAI in science communication. Similarly, systematic literature reviews, while robust for established areas of research, are best suited to large bodies of work (Grant and Booth, 2009), which is not currently applicable to the topic at hand.

To address the question of what constitutes quality science communication with ComAI, we adopted a two-step approach: We first conducted a narrative review to identify the dimensions and topics being discussed regarding quality in science communication and the role of ComAI. Second, we performed a thematic analysis to distil broader themes within these topics. This approach enabled us to respond swiftly to developments in both technology and scholarly debates while synthesising a wide range of interdisciplinary literature. By doing so, we aimed to develop a comprehensive understanding of the unique challenges and opportunities presented by ComAI in science communication.

Our narrative review followed Greenhalgh et al.’s (2005) six-phase approach. In the planning phase, we assembled a multidisciplinary team with expertise in science communication, communication science, artificial intelligence, and computer science. The search phase involved identifying relevant literature across four thematic areas. First, we examined quality in science communication using terms such as *‘science communication’ AND (‘quality’ OR ‘ethics’ OR ‘standards’ OR ‘best practices’)*, focusing on key dimensions like accuracy, inclusivity and ethical responsibility (e.g. Fähnrich et al., 2023; Medvecky and Leach, 2019; Olesk et al., 2021). Second, we explored quality in ComAI with queries such as *‘artificial intelligence’ OR ‘AI’ AND (‘quality’ OR ‘benchmarks’ OR ‘best practices’ OR ‘evaluation’)*, consulting both peer-reviewed studies and preprints (e.g. Bang et al., 2023; Johnson et al., 2023). Third, we reviewed AI governance and ethics using *‘artificial intelligence’ OR ‘AI’ AND (‘ethics’ OR ‘governance’ OR ‘trust’)*, incorporating policy documents and reports from organisations like the European Commission and Google DeepMind (European Commission, 2024; Gabriel et al., 2024). Finally, we examined AI in science communication and adjacent fields – such as journalism, science education and research – through combinations like *‘artificial intelligence’ OR ‘AI’ AND (‘science communication’ OR ‘journalism’ OR ‘science education’ OR ‘media studies’)*, integrating emerging interdisciplinary work (e.g. Bockting et al., 2023; Long and Magerko, 2020; Schäfer, 2023). We ran these targeted searches in major academic databases, including Web of Science, Scopus, Google Scholar, IEEE Xplore and arXiv. To remain responsive to new developments, we adopted a dynamic selection process, allowing us to integrate emerging research as it became available. This included having all authors contribute sources from their respective areas of expertise throughout the manuscript development, ensuring a broad and reflexive approach. By continuously refining our selection, we aimed to balance conceptual depth with adaptability to evolving debates.

In the appraisal phase, we critically evaluated sources for relevance and theoretical contribution. In the synthesis phase, we organised key insights into thematic categories that informed our analysis of quality science communication with ComAI. Finally, in the recommendation phase, we developed a principled framework for guiding the responsible use of ComAI in science communication, which is elaborated in the discussion section.

Our thematic analysis aimed to organise the various issues identified in the narrative review into broader themes. Following Braun and Clarke’s (2006) guidelines, we first familiarised ourselves with the issues, generated initial codes, and constructed potential themes. Through an iterative process of reviewing, merging and refining these themes, and defining them in relation to our conceptualisation of principles, we engaged in ongoing reflexive dialogue among team members. This process led us to construct five broad principles for quality science communication with ComAI. In the final step, we related our analysis back to our research question and literature review, producing a coherent narrative around questions of quality in science communication and ComAI. The resulting principles thematically reflect debates on quality in both science communication and ComAI and address the numerous issues surrounding the development and diverse applications of these technologies in science communication.

## 3. Principles of science communication with ComAI

The five principles (Table 1) are broad, flexible and applicable to most ComAI science communication scenarios. They follow a narrative path reflecting key stages in the process: getting the science ‘right’ (*scientific integrity*), focusing on people’s agency and needs (*human-centric communication*), ensuring science communication with ComAI does ‘good’ (*ethical responsiveness*), reaching out to diverse communicators and publics (*inclusive impact*), and managing its development and implementation (*governance*). Depending on specific contexts and goals, different principles may take precedence or conflict, requiring careful consideration and balancing.

**Table 1.** Principles of quality science communication with ComAI.

Principle	Definition	Key aspects
Scientific integrity	Upholding rigorous science communication standards	Accuracy, reliability, comprehensiveness, objectivity, verifiability
Human-centricity	Prioritising human needs and experiences	Agency, relevance, resonance, respect
Ethical responsiveness	Addressing moral implications and societal impacts	Privacy and security, beneficence, non-maleficence, justice, autonomy
Inclusive impact	Ensuring broad accessibility and representation	Representation, empowerment, accessibility, sustainability
Governance	Establishing oversight and accountability mechanisms	Transparency, monitoring and feedback, accountability, collaboration, learning

### Scientific integrity

The first principle we highlight sets the tone for our framework: Science communication with ComAI technologies must be geared towards upholding *scientific integrity*, understood as accurately representing and respecting the integrity of scientific processes and findings. As Leßmöllmann (2019) notes, this involves ‘sticking to rules of good scientific practice’ (p. 671) in how scientific information is communicated, while recognising the distinct goals and logics of science communication.

Scientific integrity, particularly in terms of accuracy and reliability, appears as a central component in existing quality frameworks for science communication (Fähnrich et al., 2023; Olesk et al., 2021; Taddicken et al., 2024). While ComAI offers unprecedented capabilities, it risks creating and disseminating inaccurate, incomplete, biased, and unverified scientific information, and potentially harming epistemic trust and the public perception of science (Fecher et al., 2023a). These technologies should avoid the ‘manipulation of facts and data’ in the ‘enterprise [of] producing reliable knowledge’ (Andorno, 2021: 93; 103) by ensuring that science communication with ComAI demonstrates accuracy, reliability, comprehensiveness, objectivity and verifiability, and is ultimately designed to maintain scientific integrity every step of the way.

#### Accuracy

Long recognised as a quality standard in science communication (Hansen, 2016), accuracy is challenged by ComAI’s inconsistent responses and potential for hallucinations. Addressing this requires rigorous validation and cross-referencing of information with algorithms capable of monitoring scientific debates and verifying claims against authoritative sources (Lewis et al., 2021). Communicators should also implement robust verification mechanisms through human-in-the-loop approaches which could ensure that the content generated by ComAI aligns with established scientific facts and prompt error correction (Demartini et al., 2020).

#### Reliability

Consistent and dependable information across contexts and time is crucial for ComAI’s use in science communication. However, these systems often produce inconsistent responses and outdated information (Zhuo et al., 2023). Addressing these issues requires implementing version control, regular updates, and cross-checking methods, all under constant human oversight to ensure alignment with current scientific understanding (Johnson et al., 2023). By prioritising reliability through these measures, ComAI can become a more trustworthy tool for consistent and up-to-date science communication.

#### Comprehensiveness

Comprehensive science communication aims to provide a complete overview of available scientific knowledge, including current consensus, uncertainties and knowledge gaps. While ComAI can synthesise information from diverse sources (Fecher et al., 2023a), it is limited by biases in scientific publishing, such as the predominance of English-language and Global North sources (Arenas-Castro et al., 2024). Designing comprehensive ComAI requires training on diverse, multilingual sources and implementing human-in-the-loop approaches to ensure balanced representation of scientific consensus and ongoing debates.

#### Objectivity

While true objectivity is debated, aiming for impartial presentation of scientific knowledge remains crucial (Olesk et al., 2021). ComAI’s potential to process vast amounts of information impartially is tempered by susceptibility to built-in biases (Ferrara, 2024). To mitigate this, algorithms should use balanced datasets and incorporate cheques against undue influence of particular viewpoints (Titus, 2024). Human oversight, such as the FairCaipi approach, offers promising avenues for bias mitigation (Heidrich et al., 2023). Diverse training datasets and transparent evaluation processes can help ensure neutral communication focused on the best available scientific evidence.

#### Verifiability

Ensuring traceability of information to original sources while minimising the hallucination of sources is crucial for maintaining ComAI’s scientific integrity (Schäfer, 2023). As such, ComAI should always integrate references to scientific information, with human verification. Transparency in data sources and methods is essential (Dijkstra et al., 2024). Moreover, referencing should be robust, including links to original sources and metadata, clear indications of paywalled content, and integration with open-access repositories (Wang et al., 2020). This empowers users to verify claims, fostering trust and scientific literacy.

### Human centricity

Science communication has long emphasised human-centred approaches for effective communication incorporating strategies like direct dialogue, ‘know your audience’, emotional messaging and active listening (Olesk et al., 2021). These approaches engage people in ways that resonate with their experiences, values and needs (Kearns, 2021).

The advent of ComAI introduces new dimensions to human-centric science communication. Advances in natural language processing have enhanced ComAI’s ability to interpret and adapt to different contexts (Gabriel et al., 2024), potentially making it a highly effective science communicator (Schäfer, 2023). Indeed, recent studies show that chatbot conversations can be persuasive (Costello et al., 2024) and that users perceive it as trustworthy (Molina and Sundar, 2022). While potentially beneficial, these capabilities raise concerns over ‘interaction risks’ such as manipulation, deception and a lack of authenticity (Weidinger et al., 2021).

As ComAI takes on more agentic roles, it is crucial to ensure that science communication retains its ‘human touch’ by designing systems that enhance rather than replace human capabilities, promote effective communication relevant to individual users, and respect human dignity and agency.

#### Human agency

AI development frameworks emphasise designing AI technologies that augment rather than replace human capabilities (Ozmen Garibay et al., 2023; Shneiderman, 2020). For science communication, this means creating ComAI tools that empower the human agency of practitioners and the public. For example, human-in-the-loop systems can ensure that humans maintain control over data selection and presentation (Mosqueira-Rey et al., 2023). ComAI also offers promising possibilities for amplifying human creativity through co-creative interactions while preserving empathy and intuition (Mollick, 2024). Science communicators can harness ComAI to enhance their capabilities and streamline workflows, opening up innovative avenues for presenting scientific knowledge (Schäfer, 2023).

#### Relevance

Addressing the needs, interests and contexts of the audience is central to ensuring that science communication is relevant and effective in achieving its goals (Wicke and Taddicken, 2020). The most advanced ComAI systems are already designed to understand and respond to the specific concerns and experiences of each user (Gabriel et al., 2024). This significantly enhances the potential for creating highly relevant and personalised science communication interactions (Schäfer, 2023). While this personalization can make scientific information more accessible and engaging, increasing its impact and reach, it also raises concerns about potential echo chambers and the loss of shared narratives as a result of excessive curation (Ozmen Garibay et al., 2023).

#### Resonance

Emotion-laden science narratives create a connection to the audience or resonance, increasing interest in scientific topics (Bilandzic et al., 2020). ComAI introduces new dimensions to this emotional aspect, as users form emotional connections ranging from fascination to perceived eeriness (Baek and Kim, 2023). ComAI can even outperform humans in emotional awareness (Elyoseph et al., 2023) and changing perspectives (Costello et al., 2024). While this allows ComAI to create inspiring and motivating content, it raises ethical concerns about potential manipulation (Weidinger et al., 2021). Balancing emotional engagement and safeguarding the agency of the public is crucial (see ethical responsiveness).

#### Respect

In science communication, respect means recognising the dignity of all participants, creating spaces for meaningful dialogue and maintaining civility (Taddicken et al., 2024). This is increasingly important, given the rise of uncivil communication towards scientists and communicators, particularly in digital spaces around controversial socio-scientific issues (Anderson and Huntington, 2017). ComAI can promote respectful communication by detecting and removing offensive language while promoting inclusive terminology (Shams et al., 2025). Furthermore, ComAI systems can be designed to model respectful discourse, recognise diverse perspectives and preserve human agency through careful curation of training data and ongoing monitoring (see section Governance).

### Ethical responsiveness

Science communication practitioners bear the responsibility not only to convey accurate information effectively but also to consider the potential impacts of their practices and messaging on individuals and society (Lamb, 2018 [2017]; Medvecky and Leach, 2019). This involves a reflective approach, contemplating the consequences of disseminating scientific knowledge, preventing harm from misinterpretation or exaggeration and respecting individuals’ choices to engage or disengage with science content, among other issues often discussed in the literature (e.g. Resnik, 2019).

ComAI introduces new ethical complexities such as privacy concerns and bias amplification (Weidinger et al., 2021), necessitating a robust framework for its responsible development and use. Ethical responsiveness ensures that the use of ComAI in science communication adheres to moral ideals and proactively addresses the unique challenges posed by this technology. It requires actively integrating ethical reflection and action into the design, deployment, governance and use of ComAI systems (Stahl and Eke, 2024). Drawing from the convergence in the AI ethics discussions (Floridi et al., 2018; Jobin et al., 2019), we focus on five key issues.

#### Privacy and security

The use of ComAI in science communication necessitates a firm commitment to both protecting user privacy and securing data against unauthorised access or misuse (Jobin et al., 2019). This requires strict data governance policies, including privacy-preserving techniques like data minimization and robust security measures such as encryption (Huang et al., 2023). Crucially, both science communicators utilising these technologies and the public engaging with it must be provided with transparent information about data usage including ComAI’s processing methods and how user interactions shape responses. All users should have meaningful control over their data, and ensuring privacy protection is integral to ComAI-enabled science communication.

#### Beneficence

ComAI’s implementation in science communication should aim to promote human well-being and positively affect audiences and users (Floridi et al., 2018). Its design and use must harness the technology’s potential for social impact, such as making scientific knowledge widely accessible and contributing to a well-informed society (Schäfer, 2023). By prioritising beneficence, science communicators can ensure that ComAI’s capabilities are leveraged for broad societal benefits.

#### Non-maleficence

Avoiding harm is central to ComAI’s use and development (Floridi et al., 2018). This involves identifying and mitigating potential risks such as misinformation spread or bias reinforcement (Zou and Schiebinger, 2018). Moreover, science communicators must be prepared to respond swiftly and transparently to any instances of ComAI-caused harm, with clear protocols for monitoring and accountability (see section Governance).

#### Justice

Science communication with ComAI should facilitate the fair and equitable impacts of communication (see Medvecky and Leach, 2019). Practitioners should work to ensure that the benefits and risks of ComAI are distributed justly across different communities, with particular attention to marginalised or vulnerable groups (Canfield et al., 2020; Dawson, 2014). This includes actively combating the potential for ComAI to amplify societal inequities like the digital divide (Luttrell et al., 2020). By centring justice, science communication can use ComAI to dismantle, rather than entrench, societal inequities (see inclusive impact).

#### Autonomy

Respecting individual autonomy (Floridi et al., 2018) means enabling informed decisions about engaging with science through ComAI. Practitioners should have the freedom to use their judgement in reviewing and approving ComAI-generated content, rather than deferring blindly to the technology. Likewise, users should be empowered to understand how ComAI shapes their access to and understanding of scientific information, and have the option to opt out without disadvantages (Del Valle and Lara, 2023). This ensures ComAI use remains a matter of informed choice rather than technological imposition.

### Inclusive impact

Science communication increasingly recognises the importance of inclusivity, aiming to engage and empower diverse communities by addressing historical and systemic barriers that have excluded certain groups from fully participating in and benefitting from science (Dawson, 2014). Inclusive science communication seeks to amplify underrepresented voices, provide equitable representation in and access to scientific knowledge and foster a sense of belonging and agency in science (Valdez-Ward et al., 2024).

ComAI presents both opportunities and challenges for inclusive science communication. While its scalability could make science more accessible for diverse audiences, there are risks of perpetuating or amplifying existing biases if not developed with inclusivity in mind (Zou and Schiebinger, 2018). Moreover, the environmental impact of large-scale AI systems raises concerns about long-term sustainability and equitable distribution of benefits and burdens (Van Wynsberghe, 2021).

Inclusive impact ensures that ComAI in science communication actively promotes diversity, equity and accessibility, both short term and long term. It requires a proactive approach that centres marginalised voices, empowers diverse users and ensures accessible and sustainable benefits for all. We propose four key issues to operationalise inclusive impact.

#### Representation

Involving underrepresented voices in shaping ComAI’s development, content, and use is crucial (Canfield et al., 2020). This includes diverse perspectives in algorithm design, training data curation, and scientific narrative framing (e.g. Katell et al., 2020). Partnering with community organisations and leveraging expertise from underrepresented backgrounds is essential (Humm and Schrögel, 2020). By centring marginalised communities’ experiences, ComAI can amplify diverse ways of engaging with science, fostering a more inclusive scientific discourse.

#### Empowerment

ComAI should foster critical consciousness and agency among diverse publics, enabling them to leverage science for personal and sociopolitical transformation (Stamboliev, 2023). This involves designing systems that encourage critical thinking, reflexivity and activism, providing tools for people to apply scientific knowledge to address the issues that matter to them. For example, ComAI applications could guide users in analysing local environmental challenges and support community-driven initiatives for developing advocacy strategies and capacity building. By creating systems and promoting uses that empower people, especially among marginalised populations (Valdez-Ward et al., 2024), ComAI can contribute to a more equitable and participatory science communication environment.

#### Accessibility

ComAI-driven science communication should be designed with a strong focus on accessibility and scalability (Ozmen Garibay et al., 2023), ensuring it can effectively reach and serve individuals across a wide spectrum of abilities, learning styles, and technological contexts (see Rocha et al., 2020). This involves considering perceptual accessibility (e.g. providing alternative formats for visual or auditory content), cognitive accessibility (e.g. offering clear, concise language), and operational compatibility (e.g. ensuring ComAI tools integrate seamlessly into existing infrastructures). Developing efficient algorithms, user-friendly interfaces and adaptable content delivery mechanisms, among other things, is crucial for inclusive impact at scale.

#### Sustainability

The long-term inclusive impact of ComAI in science communication depends on the sustainability of these systems (Ozmen Garibay et al., 2023). This involves prioritising energy efficiency and renewable resources in the deployment of ComAI, as well as supporting research into more sustainable AI architectures (Verdecchia et al., 2023). It also means investing in the long-term infrastructure needed to ensure equitable access and participation, especially in underserved communities. By embedding sustainability into ComAI development and use, science communicators can ensure its lasting, inclusive impact (Van Wynsberghe, 2021).

### Governance

Science communication increasingly acknowledges the critical role of governance structures in regulating practices and ensuring output quality (Fecher et al., 2023b). Traditionally, this has involved editorial policies, ethical guidelines, and institutional review boards, among other things, aimed at maintaining science communication standards over time in dynamic environments.

However, the complexity and opacity of advanced ComAI systems challenge our understanding and regulation of their operations. These systems often operate as ‘black boxes’, with outputs not easily interpretable even by their creators (Floridi et al., 2018). Moreover, some ComAI companies lack transparency regarding training datasets and algorithmic processes, raising concerns about biases and reproducibility, while liability structures remain unclear, especially when AI systems produce harmful or inaccurate content (Coeckelbergh, 2020; Liesenfeld et al., 2023).

As these technologies rapidly advance and integrate into the field, it becomes crucial to establish clear quality assurance mechanisms through governance. We propose five key aspects.

#### Transparency

Crucial in most ComAI governance frameworks (Jobin et al., 2019), transparency involves embracing an attitude of openness about system development and operation. This includes disclosing methodologies, data sources, and algorithmic functions used in ComAI processes (Floridi et al., 2018), as well as potential conflicts of interest, uncertainties and risks (Brundage et al., 2020). Opening this socio-technical ‘black box’ could foster trust in the quality and validity of science communication content between the involved stakeholders, and facilitate the collaborative development of these technologies.

#### Monitoring and feedback

Effective governance of these systems requires continuous monitoring and feedback mechanisms (Ozmen Garibay et al., 2023). The process should be collaborative, with feedback loops between developers, scientists, communicators, users and affected communities for tracking outputs and impacts, while facilitating corrections (Sadek et al., 2024). For instance, if ComAI over-represents data from specific regions in climate change summaries, monitoring can detect and address this bias. This approach provides continuous insights into the performance of ComAI for science communication and enables rapid response to concerns.

#### Accountability

Clear accountability mechanisms are essential for ensuring ComAI system quality. This involves delineating responsibility and liability across all parties, from developers to end-users and establishing effective processes to investigate and redress harms or failures (Coeckelbergh, 2020). For example, if a ComAI system inaccurately reports the misconduct of an academic (Verma and Oremus, 2023), clear protocols should exist for swift correction and holding responsible parties accountable. However, accountability should not be just about assigning blame, but also about ensuring that all stakeholders in science communication with ComAI are committed to this technology’s responsible development and use (Gabriel et al., 2024).

#### Collaboration

Active engagement of diverse stakeholders throughout the ComAI system lifecycle is crucial (Gabriel et al., 2024; Ozmen Garibay et al., 2023). This involves incorporating perspectives of science communicators and their audiences from development through application and evaluation. Effective governance is supported by multi-stakeholder advisory boards and participatory design processes, facilitating continuous dialogue between science communication experts, publics and AI developers (Pöhler et al., 2024; Sadek et al., 2024).

#### Adaptive learning

ComAI governance requires all stakeholders – including policymakers, science communicators, AI developers and the public – to engage in continuous learning to maintain effective oversight. This involves ongoing education to match the pace of technological evolution (Foffano et al., 2023) and regular updates to AI literacy and competency standards (Long and Magerko, 2020). By fostering a culture of adaptive learning, the governance process becomes more responsive, preparing all parties to address emerging challenges and promoting informed engagement with AI technologies in science communication.

## 4. Discussion

Our framework presents a comprehensive and systematic approach to quality science communication with ComAI. The five principles – scientific integrity, human-centricity, ethical responsiveness, inclusive impact and governance – are designed to serve as reflexive heuristics, prompting critical examination of science communication with ComAI.

Our goal is to encourage various stakeholders – science communicators, AI developers, policymakers and diverse publics – to consider crucial aspects of ComAI: how it can uphold standards of accurate, reliable and verifiable science communication; the extent to which it prioritises the needs, preferences and agency of human users; its responsible development and use; its potential to promote inclusive scientific spaces; and the effectiveness of mechanisms for ensuring its quality across changing circumstances. By reflecting on these issues, those who engage with ComAI for science communication can navigate its complexities with greater intentionality and awareness, contributing to a culture of reflective practice and continuous improvement (Roedema et al., 2022).

In the following sections, we illustrate the application of our five principles across different levels: guiding ComAI development, informing practical implementation via guidelines and competencies, establishing quality evaluation indicators, and aligning with future developments in ComAI and science communication. This multi-faceted exploration demonstrates the framework’s versatility and potential impact on the evolving landscape of science communication.

### Development

The development of ComAI technologies for science communication offers a unique opportunity to embed quality principles from the outset.

Applying the principle of scientific integrity, developers could, for instance, create ComAI systems with robust fact-checking capabilities and source attribution mechanisms (Lewis et al., 2021). This might involve algorithms that cross-reference claims against peer-reviewed literature and flag inaccuracies, or features providing clear citations and links to original research (Demartini et al., 2020). Human-centricity in development would focus on creating user interfaces and interaction models that prioritise the needs and preferences of both science communicators and their audiences (Ozmen Garibay et al., 2023). This could involve customisable AI assistants adapting their communication style based on users’ scientific knowledge or allowing easy requests for clarifications on complex topics (Gabriel et al., 2024). Ethical responsiveness could be integrated by implementing safeguards against harms and prioritising well-being, safety, privacy and justice. Examples include safeguards against misuse or manipulation of scientific information, algorithms to detect and mitigate biases or transparent systems that clearly communicate the limitations and uncertainties of ComAI-generated content (Stahl and Eke, 2024; Weidinger et al., 2021). The principle of inclusive impact could drive strategies like creating multilingual and culturally adaptive ComAI tools through representative sampling in training and testing phases (Shams et al., 2025). Another approach could leverage scalability to address the digital divide, making scientific knowledge more accessible to diverse global audiences (Luttrell et al., 2020). Finally, the governance principle could inform various design choices. These include creating built-in monitoring and feedback mechanisms, such as learning analytics dashboards to track ComAI tools’ performance in science communication (Klerkx et al., 2017), and incorporating provisions such as open APIs (Application Programming Interfaces) for third party access (Hartmann et al., 2024). Collaborative partnerships could systematically study these systems’ use and effects, ensuring ongoing improvement and alignment with science communication goals.

By anchoring the development process in these principles through approaches that involve all stakeholders, such as value sensitive design (VSD) for shaping safe AI design parameters (Sadek et al., 2024), we can create ComAI tools that push technological boundaries while upholding high-quality standards for science communication. This approach ensures that as these tools evolve, they remain aligned with the core goals of accurate, effective, ethical and inclusive science communication.

### Practice

As ComAI becomes more prevalent in science communication, practitioners need to be equipped with the skills and knowledge to use these tools effectively and responsibly. The development of ‘ComAI literacy’ as a core competency for science communicators will be essential. While existing AI literacy frameworks focus on broad AI concepts and technical specifications (Long and Magerko, 2020), ComAI literacy should address the unique challenges and opportunities presented by these technologies, including practical applications and citizen empowerment (Stamboliev, 2023).

The five principles of quality in science communication with ComAI proposed in this article provide a foundation for a comprehensive ComAI literacy framework tailored to the needs of science communicators. This framework would encompass a range of interconnected competencies. At its core, scientific integrity would guide practitioners in verifying AI-generated content and understanding the limitations of ComAI in scientific accuracy. Building on this, human-centricity would work around developing skills in designing user-friendly ComAI interfaces and interpreting AI outputs in ways that resonate with audiences. As science communicators navigate the ethical landscape of ComAI, the principle of ethical responsiveness would foster their ability to recognise and address potential dilemmas. The inclusive impact principle would shape competencies in adapting ComAI applications for diverse audiences and identifying hidden biases, ensuring that the benefits of these technologies reach all segments of society. Finally, governance could help develop competencies in understanding and contributing to ComAI regulations and policies. By structuring ComAI literacy around these principles, science communicators can develop a well-rounded skill set that addresses the multifaceted challenges of working with these technologies.

The principles of quality in science communication with ComAI can also contribute to the creation of professional guidelines (Dijkstra et al., 2024; Henke, 2024). By embedding these principles in codes of conduct, best practice recommendations or certification programmes, the field can establish norms for the use of ComAI in science communication. For example, guidelines based on scientific integrity could establish standards for fact-checking ComAI-generated content, while those informed by ethical responsiveness could provide guidance on mitigating potential biases. The principles of governance and inclusive impact could support the inclusive development of these guidelines, ensuring that different perspectives are considered. Importantly, the development of such professional guidelines should go hand in hand with building ComAI literacy: By integrating these guidelines into training programmes, practitioners can develop the skills to apply and shape these guidelines in their work.

### Evaluation

The third application of our principles relates to evaluating the quality of science communication with ComAI, to ensure that these systems serve their intended purposes and benefit both science communicators and the diverse publics they engage. Our five principles could provide a basis for evaluating different aspects of ComAI content, use and engagement in science communication contexts.

For example, the principle of *scientific integrity* could guide the development of measures to assess the accuracy, completeness and verifiability of scientific information presented by ComAI systems, for example, by using natural language processing and machine learning algorithms to verify claims against reputable scientific sources and to flag potential inaccuracies, inconsistencies or hallucinations (e.g. Min et al., 2023). In addition, the principle of *human-centricity* emphasises the importance of considering the needs of users and the contexts in which ComAIs are deployed. This could be assessed through metrics that measure user engagement, satisfaction, and the value of ComAI in meeting the needs and preferences of different audiences, for example, by combining user surveys, interviews and the analysis of behavioural data to track how users interact with ComAI in science communication and their attitudes towards it (e.g. Abdaljaleel et al., 2024). Moreover, the principles of *ethical responsiveness* and *inclusive impact* underscore the need for evaluation frameworks that assess the broader societal implications of ComAI in science communication. This could include examining specific issues such as privacy, security and the risks associated with the perpetuation or exacerbation of existing biases and inequalities. Evaluation methods could include audits of ComAI systems for security, bias and inclusivity (Hartmann et al., 2024), as well as longitudinal studies that track the long-term impact of ComAI on public understanding, attitudes and engagement with science (e.g. Polyportis, 2024). Finally, the *governance* principle could be used to evaluate the mechanisms for monitoring, feedback and collaboration. This could include assessing the transparency and accountability of monitoring processes, the responsiveness of ComAI systems to user feedback and the level of stakeholder diversity and engagement in their development and governance.

While further research and collaboration between science communicators, AI developers, and other stakeholders will be needed to refine our principles and translate them into concrete evaluation measures, they represent a modest first step towards operationalizing quality indicators for science communication with ComAI and ensuring that ComAI technologies are developed and deployed in ways that genuinely improve the quality and impact of science communication.

### Outlook

ComAI technologies are poised to profoundly influence science communication in the coming years (Schäfer, 2023), with their rapid advancement suggesting that today’s iterations may be the least sophisticated we will ever see (Mollick, 2024). As these systems become increasingly accurate, reduce hallucinations, and improve their ability to pass as human-like entities (Gabriel et al., 2024), their impact on how scientific information is accessed, processed and communicated will likely grow exponentially (Henke, 2024). Our principles were constructed with this future in mind, acknowledging that ComAI could become a ubiquitous presence in scientific discourse.

From current applications like ChatGPT and Perplexity AI, to potential future developments enabling seamless interactions with virtual scientists, ComAI has the power to democratise scientific knowledge while also introducing significant risks. The task ahead lies in balancing the expanding capabilities of ComAI with the core goals of science communication including scientific accuracy, human-centric engagement, ethical conduct, inclusivity and responsible oversight. Ongoing critical reflection and adaptation of these principles will be crucial to ensuring that ‘good’ science communication not only survives but thrives in the age of AI.

## 5. Conclusion

Our approach offers strengths in interdisciplinary insights and adaptability to various contexts. However, limitations exist. The narrative review may have overlooked nuances that more structured methodologies might capture, and our definition of quality, while comprehensive, may not encompass all dimensions. The broad nature of principlism, while flexible, may pose challenges for immediate implementation. In addition, our perspective as academics at European universities may limit the breadth of views included.

Despite these limitations, our work provides a solid conceptual foundation for future research and practice. Empirical validation of these principles through expert surveys and case studies will be crucial. Similarly, there is a pressing need to translate these broad concepts into actionable guidelines for different contexts within science communication. Interdisciplinary collaboration among science communicators, AI developers, ethicists, policymakers and other stakeholders will be central to refining and implementing these principles. Moreover, we call for broadening perspectives beyond our Western, academic viewpoint to develop a truly global framework.

As ComAI technology rapidly evolves, our principles aim to promote critical discussions about its role in science communication and shape governing normative frameworks. Ongoing research, collaboration and critical reflection are essential to harness the potential of ComAI while safeguarding the integrity and impact of science communication. We call on researchers, practitioners, and policymakers to engage with these principles and contribute to shaping the future of science communication in the age of AI.

## Notes

1. While principlism traditionally addresses ethical decisions in bioethics (Beauchamp and Childress, 2001), we extend it to ‘quality’ in science communication. This approach builds on previous attempts to develop principles in science communication that link ethics with effectiveness (Medvecky and Leach, 2019). Similarly, in medical practices, ‘quality principles’ have been paired with ‘ethics principles’, recognising their essential intersection (Greenhalgh et al., 2021).

2. The concept of ‘principle’ lacks a universal definition, with varying perspectives on ethical frameworks’ functions and obligations. These differences often relate to commitments to meta-ethical theories like Deontology or Utilitarianism (Davis, 1995). Following Medvecky and Leach (2019), we view principlism as a balanced approach between rigid meta-ethical theories and flexible casuistry, allowing for adaptable yet principled decision-making.
